# Resting muscle tension and trigger points in elite junior short-track athletes and healthy non-athletes: a cross-sectional examination

**DOI:** 10.3389/fspor.2024.1412412

**Published:** 2024-08-16

**Authors:** Mariusz Konieczny, Jakub Matuska, Paweł Pakosz, Przemysław Domaszewski, Marta Skulska, Pablo Herrero, Elżbieta Skorupska

**Affiliations:** ^1^ Faculty of Physical Education and Physiotherapy, Opole University of Technology, Opole, Poland; ^2^ Poznan University of Medical Sciences, Doctoral School, Department of Physiotherapy Poznan, Poznan, Poland; ^3^ Unit of Histology and Neurobiology, Department of Basic Medical Sciences, Faculty of Medicine and Health Sciences, Rovira I Virgili University, Reus, Spain; ^4^ Department of Health Sciences, Institute of Health Sciences University of Opole, Opole, Poland; ^5^ Faculty of Health Sciences, IIS Aragon, University of Zaragoza, Zaragoza, Spain; ^6^ Department of Physiotherapy, Poznan University of Medical Sciences, Poznan, Poland

**Keywords:** myofascial pain, electromyography, trigger point, elite athletes, short-track

## Abstract

**Introduction:**

Short-track speed skating (short track) is an Olympic sport characterized by a specific body position and counterclockwise movement on the track. Based on previous studies, we hypothesized that athlete body positions employed in this sport could lead to asymmetric overuse disorders of the left lower limb muscles. An increased number of latent trigger points (LTrPs) was confirmed in individual muscles of the overloaded left limb of short-track athletes. This study aimed to compare the number of LTrPs and the level of resting muscle tone between elite junior short-track athletes and healthy non-athletes.

**Methods:**

The experimental (EXP) group comprised 15 elite short-track junior athletes from the Polish national team and the control (CON) group comprised 15 healthy young volunteers. In both groups, the left leg was tested for (i) the presence of LTrPs and (ii) resting muscle tone (RMT), assessed using surface electromyography in six muscles.

**Results:**

The EXP group showed a higher number of LTrPs in the left lower limb, compared with the CON group. The muscle that was most significantly affected in the athletes was the vastus lateralis obliquus [*χ*^2^ (1, *N* = 30), *p* < 0.001, V Cramer = 0.71]. This muscle also differed significantly between the groups in terms of the RMT (*p* = 0.033, Cohen's d = 0.87).

**Conclusions:**

Elite short-track junior athletes presented with increased RMT and an increased number of LTrPs in the vastus lateralis oblique muscle, compared with healthy non-athletes.

## 1 Introduction

Short track is an Olympic sport characterized by a specific body position and counterclockwise movement on the track. Previous studies indicated that athletes’ body positions and movements can lead to asymmetric overuse of the lower limb muscles (1, 2). This is supported by evidence demonstrating the asymmetric overload of the limbs, usually on the left side, in aspects such as muscle desaturation and neuromuscular profile myelopathy (3, 4). Muscle fatigue on the left side of the body appears to be common among elite junior short-track athletes, likely due to the nature of the activity, as the movement is always counterclockwise (4) or due to a lack of experience in skating techniques (2). Furthermore, short-track athletes have asymmetric fatigue-related abnormalities in the gluteal muscles, which affect their posture (1, 2, 5). Stoter et al. (6) showed that the bioelectrical activity of the muscles of speed skaters is correlated with speed on different sections of the track; however, they did not analyze whether muscle asymmetry and other disorders influenced this phenomenon. One possible explanation for changes in muscle bioelectrical activity may be trigger points (TrPs). TrPs are hyperirritable areas within the taut band that can be divided into latent (no spontaneous pain, elicited only by irritation) and active (causing spontaneous pain, which is actually responsible for pain symptoms). The prevalence and distribution of TrPs vary depending on the athlete's sport (7). Furthermore, TrPs can cause motor alterations e.g., changes in the muscle activation patterns or limited muscle strength (8). Efforts have been made to analyze the relationship between changes in muscle bioelectrical activity and the occurrence of TrPs (9). It has also been shown that there is an increase in the bioelectric activity of muscles near LTrPs compared to muscles without TrPs (10). It has also been shown that there is a decrease in the resting value of muscle bioelectric activity after therapy conducted on TrPs (10, 11). Nevertheless, there remains a knowledge gap regarding the prevalence of LTrPs in elite junior short-track athletes and their impact on abnormal muscle bioelectrical activity, which can directly translate into impaired movement patterns.

Although sEMG is a non-invasive method for detecting signs of muscle fatigue or muscle reaction time (4–6), it has not yet been used to detect the influence of LTrPs on muscles among athletes. Some authors (10, 11) hypothesize that one of the causes of fatigue or muscle weakness is the increased number of LTrPs; however, the diagnostics for this are not fully objective or described.

Our study aimed to compare the number of LTrPs and level of resting muscle tone between elite junior short-track athletes and healthy non-athletes.

## 2 Material and methods

This observational cross-sectional study was conducted in June 2023 following the Declaration of Helsinki and was approved by the Ethics Committee of Poznan University of Medical Sciences (Resolution No. 110/22 of March 10, 2022; Trial registration: 20/07/2022, Trial Id: ACTRN12622001016729). Anonymity was ensured by coding all data to avoid any intentional or unintentional bias and to ensure a high level of evidence. Data from the participants were anonymized and stored according to official data protection regulations (on the server of the Opole University of Technology).

### 2.1 Participants

**Figure 1:**
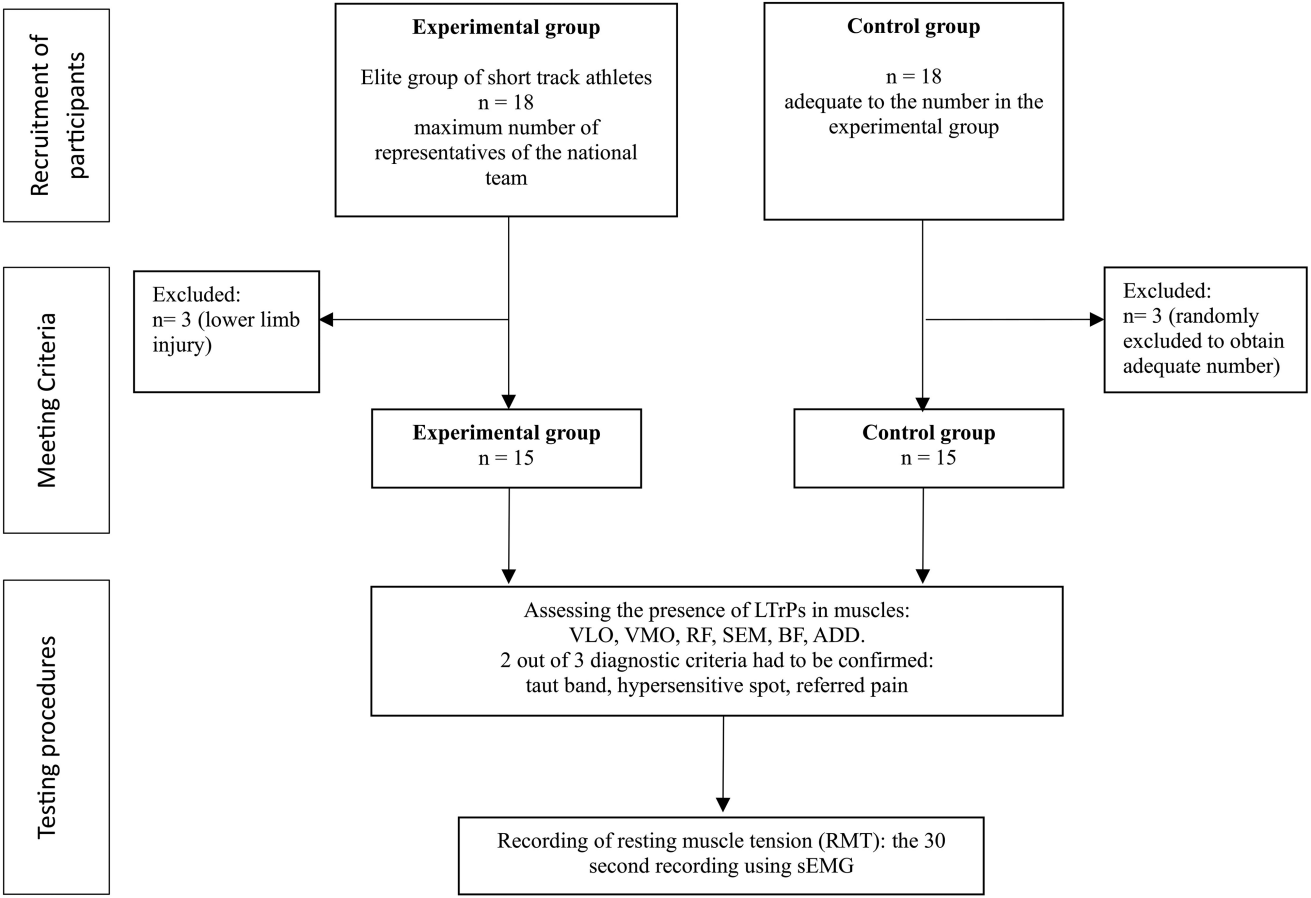
Flow diagram of the study design.

A convenience sample, comprised of all elite junior short-track athletes (*n* = 18) from the Polish national team, was included as the experimental group (EXP), and an equal number of healthy participants were recruited to form the control group (CON). In accordance with the eligibility criteria, 3 subjects were excluded from the EXP group due to lower limb injury. To obtain an equal number of participants in the control group, we randomly excluded 3 subjects. The study included 15 elite junior short-track athletes and 15 asymptomatic non-athletes. Athletes who met the following criteria were included in the EXP group: (1) those with at least 10 years of competitive experience and (2) those participating in normal training in the Polish national short-track team. The only exclusion criterion was the presence of any injuries that impeded the athletes from training at the time of the study. For the CON group, the only inclusion criterion was not having been trained on a short track, and the exclusion criterion for this group was the same as that for the experimental group—the presence of any lower limb injuries. All participants provided written informed consent to participate in this study. For minors, consent was obtained from their legal guardians. A flowchart illustrating the study design is shown in Figure 1.

### 2.2 Testing procedure

All participants were assessed to check if they met the inclusion criteria. The participants were then examined by a specialist for the presence of latent trigger points (LTrPs) in the following muscles of the left lower limb: vastus lateralis oblique (VLO), vastus medialis obliquus (VMO), rectus femoris (RF), semitendinosus (SEM), biceps femoris (BF), and adductor longus (ADD). Finally, the bioelectrical activity of the muscles was measured using sEMG by another investigator. The analysis of previous research indicates that, in short-track athletes, the above-mentioned muscles are particularly susceptible to overload owing to their position during skating. Studies have shown that in the area of the analyzed muscles, there is increased hyperemia and an increased number of TrPs during skating (2–4). All investigators of the research groups–those carrying out examinations of TrPs, those conducting sEMG, and those performing data analysis–were blinded to the group allocation. However, it is possible that TrPs and sEMG assessors can recognize athletes because of their body builds.

### 2.3 Examination of LTrPs

In this study, the presence of LTrPs was diagnosed by a myofascial pain therapist with 10 years of experience, in accordance with the international consensus on diagnostic criteria, which proposed that at least two of the following criteria must be present for LTrP diagnosis: a taut band, a hypersensitive spot, and referred pain (12). The positions of the participants during assessment were consistent with those described by Travell and Simons (13).

### 2.4 sEMG examination and signal analysis

A 16-channel sEMG system (NORAXON DTS) was used to record the signals at a sampling rate of 1,500 Hz. Signal processing was performed using NORAXON MR-XP 1.07 Master Editions software. This equipment has been demonstrated to be a valid and reliable tool for measuring sEMG activity (14). The skin was cleaned to prepare the muscles for analysis, and electrodes (Ag/AgCl) were applied according to the SENIAM method [the SENIAM project—Surface ElectroMyoGraphy for the Non-Invasive Assessment of Muscles—is a European concerted action in the Biomedical Health and Research Program (BIOMED II of the European Union)] (15, 16).

Resting muscle tone (RMT) was recorded for 30 s by a trained specialist and then subjected to root mean square processing using NORAXON MR-XP 1.07 Master Edition software. The mean value of the RMT from the analyzed sEMG recordings and the number of TrPs were subjected to statistical analysis. During the resting muscle tension test, the participant was supine and relaxed on a couch, following the SENIAM guidelines.

### 2.5 Statistical analysis

The LTrPs and RMT were statistically analyzed, as described below:

• Statistical analysis of the LTrPs: Pearson’s chi-square test (*χ*^2^) of independence was conducted to statistically compare the number of LTrPs in each muscle between the EXP and CON groups. The Cramer’s V coefficient was also calculated for each variable.
• Statistical analysis of the RMT: The Shapiro–Wilk test was conducted to check the normality of the data. Welch’s T-test was used for the parametric variables: VLO and VMO. The Mann–Whitney U test was used for non-parametric variables: RF, SEM, BF, and ADD. The mean and standard deviation of each variable were calculated. The effect size was also calculated depending on the test used [standardized effect size—d (Cohen’s d) or Glass rank biserial correlation coefficient—r].

The data were analyzed using StatsCloud software (https://statscloud.app/beta/).

## 3 Results

In the EXP group (men aged 18.5 ± 1.5 years), the abundance of LTrPs in the muscles varied and the highest number occurred in the VLO muscle, significantly distinguishing this muscle in this group. In the CON group (men aged 19.5 ± 1.6 years), LTrPs did not occur in any of the muscles studied. Pearson's chi-square test analysis showed a significant association between the predictor variable (group, EXP vs. CON) and the outcome variable (muscle, VLO): *χ*2 (1, *N* = 30), *p* < .001, Cramer's V = 0.71. However, no significant relationships were detected between the same the predictor variable (group, EXP vs. CON) and the remaining outcome variables (muscles: WMO, RF, SEM, BF, and ADD) (Table 1).

**Table 1:** The RMT, number of participants with lTrPs, and group differences in each parameter.

Predictor variable	Outcome variable	LTrPs predictor variable EXP vs. CON				RMT predictor variable EXP vs. CON		
		Yes^f^	No^f^	*p* ^a^	V Cramer	*X* ± SD	*P* ^b^ ^,^ ^c^	Effect size^d^^,^^e^
EXP	VLO	10	5	<.001^a^	0.71	2.88 ± 1.03	0.033^b^	0.865^d^
CON		0	15			2.15 ± 0.63		
EXP	VMO	3	12	0.068^a^	0.33	2.15 ± 0.63	0.885^b^	0.043^d^
CON		0	15			1.77 ± 0.31		
EXP	RF	1	14	0.309^a^	0.19	1.79 ± 0.32	0.178^c^	0.125^e^
CON		0	15			1.93 ± 0.66		
EXP	SEM	2	13	0.143^a^	0.27	1.91 ± 1.04	0.818^c^	0.246^e^
CON		0	15			1.87 ± 0.36		
EXP	BF	3	12	0.068^a^	0.33	1.75 ± 0.31	0.165^c^	0.254^e^
CON		0	15			1.91 ± 0.64		
EXP	ADD	0	15	0.032^a^	0.39	2.53 ± 1.28	0.633^c^	0.087^e^
CON		0	15			2.43 ± 0.98		

VLO, vastus lateralis obliquus; VMO, vastus medialis; RF, rectus femoris; SEM, semitendinosus; GMD, gluteus medius; BF, biceps femoris; ADD, adductor longus; Yes (TrPs), No (TrPs); RMT, resting muscle tone; TrPs, trigger points.

^a^ Pearson's chi-squared test.

^b^ T-Welch test.

^c^ Mann–Whitney *U*-test.

^d^ Standardized effect size—d (Cohen's d).

^e^ Glass rank biserial correlation coefficient—r.

^f^ Participants with TrPs (predictor variable: *N* = 30).

**Figure 2:**
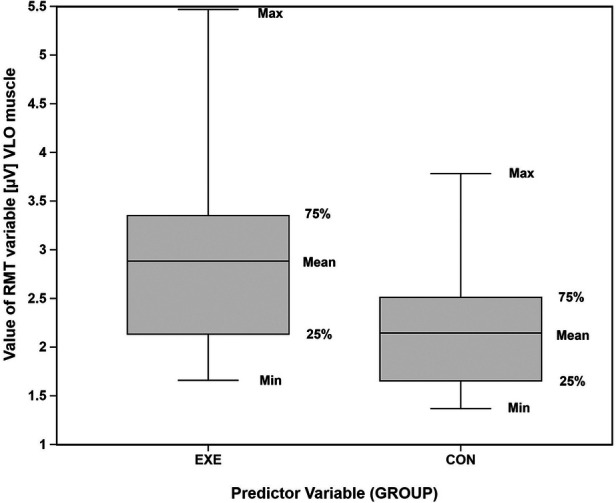
Graphic representation of the resting muscle tone in the VLO muscle between the two groups examined. Box plot of RMT values (*μ*V) in the VLO muscle for the experimental (EXE) and control (CON) groups. Boxes show the 25th-75th percentiles, lines inside the boxes indicate medians, and whiskers represent the range.

Statistical analysis of the RMT showed significant differences between the groups only in the VLO muscle. The EXP group demonstrated higher RMT for the VLO muscle (2.88 ± 1.03), compared with the CON group (2.15 ± 0.63) (Figure 2). The group comparison analysis of the RMT for the VLO, using Welch's *t*-test, showed a *p*-value of 0.333 and Cohen's d = 0.87. The other muscles assessed showed no significant differences in the RMT between the two groups.

## 4 Discussion

To the best of our knowledge, this is the first study to demonstrate the presence of distinct abnormalities in muscle bioelectrical activity in the VLO of the lower limb overloaded by the short track technique. Our study's objective was to compare the number of LTrPs and the level of RMT between elite junior short-track athletes and healthy non-athletes. The higher value of muscle bioelectrical activity observed may be attributable to an increased number of TrPs. The elite junior short-track group exhibited a higher number of LTrPs in the left lower limb, with the VLO being the most significantly affected muscle. Additionally, there was a significant intergroup difference in the RMT of this muscle.

Our results suggest that higher bioelectrical activity at rest is associated with the presence of LTrPs. An elevated resting muscle tone can cause muscle disorders and lead to increased muscle fatigue, which is of great significance for athletes. Although numerous scientific analyses have studied the bioelectrical activity of muscles associated with the presence of TrPs, most have focused on the disorders they can cause (such as their impact on strength or fatigue in specific regions) rather than comparing resting bioelectrical muscle activity.

In a study conducted by Ge et al. (10), the authors also demonstrated increased functional sEMG muscle bioelectric activity near LTrPs compared with those near non-TrPs in healthy individuals. Studies have shown that TrPs in limb muscles can affect movement kinematics, cause vasodilatation, and alter muscle bioelectrical activity parameters (17, 18). This can potentially result in muscle fatigue occurring four times faster, as measured using sEMG (1, 10). Previous studies (19) have shown that TrPs influence disturbances related to the bioelectrical activity of muscles and that there are topographic changes in the mean sEMG and peak sEMG amplitude, which indicates that individuals with TrPs exhibit a shift in muscle activity distribution, compared with asymptomatic individuals. Other authors (20) have also found increased resting-state muscle bioelectrical activity at the site of the identified LTrPs, which decreased by 23% after therapy, indicating changes in bioelectric activity in the presence of LTrPs. These studies confirmed that LTrPs generate heightened muscle tension and highlighted the need to undergo physiotherapeutic interventions independently if the treatments are more or less specific for TrPs. The results of our study indicate that elite junior short-track athletes have a higher prevalence of TrPs in the VLO muscle and elevated RMT than do healthy non-athletes. Although we did not conduct a correlation analysis, it seems probable that LTrPs affect the RMT, especially, as we observed differences in both the RMT and prevalence of LTrPs between groups solely in VLO. The occurrence of TrPs is significant not only in the development of RMT-related disorders and its direct impact on muscle fatigue (2) but also in the emergence of pain symptoms, as confirmed in other groups of athletes (21). Sánchez-Infante et al. (11) found that a specific therapeutic intervention in TrPs (i.e., dry needling) was effective in reducing elevated resting electromyographic activity in the TrP area. The effectiveness of TrP therapy on decreasing elevated bioelectrical tension was also confirmed by Schneider (22), who demonstrated a significant correlation between the effects of dry needling therapy and reduction in muscle tension in the area of TrP localization. Furthermore, the study results on the number of LTrPs in the VLO are in line with those of previous research, which concluded that short-track athletes exhibited a greater number of TrPs in the muscles of the pelvic girdle and lower extremities on the left side of the body, with a significantly higher count compared with the control group (2). This may be a distinctive feature of short track athletes. Park et al. (7) supported our findings with their outcomes, in which they demonstrated different distributions of TrPs in college athletes of various sports. Our study provides important insights that may contribute to the development of injury prevention and physical therapy programs.

One of the strengths of our study is that there has been no previous study of this kind on professional junior short-tracker athletes and that it showed a higher prevalence of LTrPs in the VLO muscle. We acknowledge that the primary limitation of such studies is the subjective diagnosis of TrPs, which remains a matter of discussion. Future studies should consider the use of thermography or ultrasonography to provide additional information for the diagnosis of muscle disorders in short-track athletes. The authors are also aware of the limitations related to the sample size, as well as the reduced sample size, which impedes the generalization of the results. However, it should be emphasized that the number of national short-track team athletes was small and we aimed to recruit the same number of individuals in the CON group.

Although further investigation with larger sample sizes is necessary and the results from this study can only be translated to a specific population of elite junior short-track athletes, this study has demonstrated the existence of an association between increased RMT and increased number of LTrPs in the VLO muscle in this population.

### 4.1 Practical applications

The results of this study can help physiotherapists of the national team to implement programs to prevent musculoskeletal disorders through the treatment of LTrPs. Physiotherapists and athletes should consider prophylactic treatment of LTrPs. Although LTrPs do not cause pain, they indicate a change in the bioelectrical activity of the muscles. The role of TrPs in musculoskeletal injuries has been overlooked or ignored for many years, and the World Health Organization has published guidelines recommending TrP management for chronic primary low back pain. However, the first draft of the 11th International Classification of Diseases has stated that muscles with TrPs can be a possible reason for chronic primary back pain with/without radiation to the lower limb. Based on these recent recommendations and the results of this study, we recommend the diagnosis and follow-up of LTrPs to prevent musculoskeletal disorders.

## Data Availability

The raw data supporting the conclusions of this article will be made available by the authors, without undue reservation.
